# Supplementary material for: The genome sequence of the Orange Beauty, Commophila aeneana (Hübner, 1799–1800) (Lepidoptera: Tortricidae)
Source: Wellcome Open Res. 2025 Sep 8;10:489. [Version 1] doi: 10.12688/wellcomeopenres.24865.1 (PMC12501591; doi:10.12688/wellcomeopenres.24865.1)
Supplement: Peer review history

# Reviewer report: Reviewer response for version 1

**DOI:** 10.21956/wellcomeopenres.27389.r133409

**Published:** 2025-10-10

**Authors:** Xin Liu

**Affiliations:** 1 Beijing Genomics Institute, Beijing, China

David C. Lees’s article provides a clear description of the genome sequencing, assembly, and evaluation of the Orange Beauty, *Commophila aeneana*. The methodology is well-documented, and the tools and software used are consistent with those commonly applied in similar genomic projects. The resulting genome data are already accessible in public databases. As a data note, I believe this article is already of high quality and presents no major issues. However, I have two suggestions for the author to consider:

 1. I noticed a significant size difference between the two haplotypes. Based on the data in Table 3, it appears that the second haplotype assembly may lack the ZW sex chromosomes. The current description of the assembly methods makes it difficult to understand why one haplotype includes both sex chromosomes. I recommend discussing the possible reasons for the length discrepancy between the two haplotypes and, in the sections on chromosome anchoring and final assembly, clarifying how the two sex chromosomes and haplotype data were ultimately integrated into the assembly of haplotype 1.

 2. In the Data Availability section, the authors mention RNA-seq and gene annotation. I believe that for a genome dataset to be considered complete—especially for downstream applications—it should include gene annotation results. Even if the available RNA-seq data are not yet comprehensive, I suggest including a preliminary gene annotation in this version of the dataset, following the standard workflows used in similar genomic studies. Further updates to the annotation can be made as more RNA-seq data become available.

Are sufficient details of methods and materials provided to allow replication by others?

Yes

Is the rationale for creating the dataset(s) clearly described?

Yes

Are the datasets clearly presented in a useable and accessible format?

Yes

Are the protocols appropriate and is the work technically sound?

Yes

Reviewer Expertise:

genomics, bioinformatics

I confirm that I have read this submission and believe that I have an appropriate level of expertise to confirm that it is of an acceptable scientific standard.

# Reviewer report: Reviewer response for version 1

**DOI:** 10.21956/wellcomeopenres.27389.r133404

**Published:** 2025-10-06

**Authors:** Jonathan Badger

**Affiliations:** 1 National Cancer Institute, Misrata, Libya; 2 National Cancer Institute, Bethesda, MD, USA

This is a straightforward and well written genome sequencing manuscript with adequate description of methods.

 It might be useful for a reader for a phylogenetic tree of related insects to be included to get a sense of where *C.* *aeneana *fits in, but I suppose this is not absolutely necessary.

Are sufficient details of methods and materials provided to allow replication by others?

Yes

Is the rationale for creating the dataset(s) clearly described?

Yes

Are the datasets clearly presented in a useable and accessible format?

Yes

Are the protocols appropriate and is the work technically sound?

Yes

Reviewer Expertise:

I am a computational biologist with a focus on genomics, particularly microbial genomics, although I have also worked in insect genomics.

I confirm that I have read this submission and believe that I have an appropriate level of expertise to confirm that it is of an acceptable scientific standard.

# Reviewer report: Reviewer response for version 1

**DOI:** 10.21956/wellcomeopenres.27389.r132174

**Published:** 2025-09-17

**Authors:** Nicola J Nadeau

**Affiliations:** 1 The University of Sheffield, Sheffield, UK

There is a good level of background information provided on the species. The methods are clear. The genome is clearly of excellent quality, having 2 fully assembled haplotypes and both sex chromosomes.

 There are some minor points that could be improved/clarified:

 Hi-C library preparation: Were multiple libraries prepared? If so, how many? If a single library then this should be clarified and specific details of the methods for that library included (eg: the number of PCR cycles).

 There are some inconsistencies about whether the second haplotype is assembled to chromosome or scaffold level. Most of the text and tables suggest it is assembled to chromosome level but “assembly statistics” text section says scaffold level.

 The Fig 6 (blob plot) text is a bit small and hard to read

Are sufficient details of methods and materials provided to allow replication by others?

Yes

Is the rationale for creating the dataset(s) clearly described?

Yes

Are the datasets clearly presented in a useable and accessible format?

Yes

Are the protocols appropriate and is the work technically sound?

Yes

Reviewer Expertise:

Evolutionary genetics and genomics. Lepidopteran ecology and evolution.

I confirm that I have read this submission and believe that I have an appropriate level of expertise to confirm that it is of an acceptable scientific standard.
